# Baltimore Medical and Surgical Society

**Published:** 1878-03

**Authors:** 


### BALTIMORE MEDICAL AND SURGICAL SOCIETY.

A case which showed either the easy communicability of the autochthonous origin of scarlet fever, was related by Dr. Lynch: In November, 1877, a man sustained a comminuted fracture of tibia and fibula of left leg. The injury was the result of a fall from an upper floor of the State Tobacco Warehouse. The limb was suspended in Smith's anterior splint; in order to obtain better extension, Coskery's splint was afterwards applied. After five weeks the man was attacked by scarlet fever of a severe form, all the characters being well marked. Albuminuria accompanied, and repeated desquamation of the skin followed the fever. No other case of scarlet fever in the house, or immediate neighborhood. After recovery from the scarlet fever, the patient had an attack of pneumonia, to which he succumbed. At the *post mortem*, the peritoneum was found deeply pigmented, almost black in places, showing precedent diffused peritonitis. Strange to say, however, the patient never complained of abdominal pain. The visceral and parietal layers of the peritoneum were firmly adhered to each other.

Dr. Evans related the following case: A young woman had for a week vomited everything taken into the stomach. Had formerly had similar spells; no fever. There was a noticeable depression of the epigastrium, "as if the stomach was absent." Distinct pulsation could be felt in the region. Nothing will relieve the vomiting except morphia, and this does so only so long as the patient abstains from eating and drinking. The Doctor was disposed to ascribe the symptoms to an aneurism of the abdominal aorta.

Dr. Thomas R. Brown related a case of fracture of the scull: About the middle of December, 1877, a drayman was struck upon the head by a stone, fracturing the skull. He fell from his dray, and was carried home in an unconscious condition; he recovered his consciousness during the night, and next day came to the out patient department at the city hospital. The fractured bone was depressed, and through the cleft the brain could be seen pulsating. There were no symptoms of cerebral disturbance. The patient was admitted to the hospital, the wound closed with sutures, and cold water dressing applied. In two weeks he was discharged well, and has so remained. The case was reported to show that energetic surgical interference is not necessary in head injuries, unless symptoms of compression are present. Drs. Lynch, Bates and Morris related cases of similar injuries, where like happy results were obtained by adopting the plan of non-intervention.

Dr. Leonard had noticed in cases of cerebral congestion, and of traumatic meningitis recently under his care, considerable diurnal variations in temperature, simulating remittent fever. He had not been able to find mention of this peculiarity in any work he had consulted.

Dr. A. B. Arnold related a case which he considered one of epileptiform neuralgia, (Trosseau). A man forty years of age, has had for eleven years left-sided facial neuralgia, particularly about the forehead and eyes. The pain is intense. The attacks come on suddenly, and last not over ten minutes; frequently not more than five. The periods of the attacks are irregular. Within the last year and a half other symptoms have made their appearance. Attacks of acute delirium, lasting from ten to fifteen minutes, come on at irregular intervals, sometimes as often as three or four times a day, at others, not over two or three times a week. Like the paroxysms of pain, these attacks of delirium come on without warning. If the patient is engaged in conversation, he all at once stops speaking, bows his head, and begins humming a tune; he does not sing, does'nt articulate—merely hums. If interrupted he flies into a violent passion. This mental aberration passes off as suddenly as it came on, and the individual resumes the conversation where it was interrupted. The paroxysms are all alike; the monotony is never varied. In consequence of his affection, the patient is unfitted for business. The prognosis is bad, as the disease is incurable. Dr. Arnold believes the cause to be intracranial.

As to treatment, nearly everything has been tried. The patient has taken "pounds" of bromide of potassium. The other bromides, belladonna, gelsemium; all the narcotics, in fact, had been tried, and seem but to aggravate the suffering. He has had tonics without avail. Hypodermic injections of morphia relieved the pain during the paroxysms, but did not prevent their occurrence. This was the only remedy relied on at present.—*Maryland Medical Journal.*

